# Self-relevance dynamically biases exploration and exploitation in value-based decisions

**DOI:** 10.1007/s00426-026-02402-6

**Published:** 2026-10-16

**Authors:** Esther S. Selvaraj, Marius Golubickis, Siobhan E. Caughey, Parnian Jalalian, Yadvi Sharma, Srishti Khasa, Leoni S. Masroujah, C. Neil Macrae

**Affiliations:** 1 School of Psychology, King’s College, University of Aberdeen, Aberdeen, AB243FX Scotland, UK; 2 Department of Cognitive Sciences, United Arab Emirates University, Al Ain, UAE; 3 Alliance Manchester Business School, University of Manchester, Manchester, England, UK; 4 Department of Psychology, University of Cambridge, Cambridge, England, UK

**Keywords:** Self, Decision-making, Exploration-exploitation dilemma, Uncertainty, Bandit task

## Abstract

While adaptive decision-making requires balancing exploration and exploitation, dominant accounts often treat this trade-off as independent of social context. Drawing on theories of self-function and motivated cognition, we tested whether deciding for oneself versus others alters exploration-exploitation dynamics. Across two experiments, participants completed a restless multi-armed bandit task for themselves or a close friend under gain (Experiment 1) and loss (Experiment 2) conditions. Results indicated that self-other differences in choice behavior emerged dynamically over time rather than reflecting a static bias. An additional computational modeling analysis revealed that self-relevance was associated with increased sensitivity to value differences (i.e., inverse temperature), consistent with more deterministic choice (i.e., exploitation). Taken together, these findings demonstrate that self-relevance does not merely influence learning but dynamically shapes decision policies over time. More broadly, the results highlight the importance of motivational context in regulating how individuals balance information gain and reward during value-based decisions.

When choosing where to eat, whom to date, or how to get home from work, decision makers face a recurring conundrum. Should they persist with options that have been successful in the past or pursue alternatives that may yield better outcomes, such as tastier meals, more engaging conversations, and shorter journeys? This trade-off—known as the exploration-exploitation dilemma—captures the tension between immediate reward and information gain and has been formalized within reinforcement-learning frameworks (Cohen et al., 2007; Sutton & Barto, 1998). In uncertain environments, these strategies serve distinct psychological functions. Exploitation maximizes expected reward, but affords limited opportunity for learning, whereas exploration entails greater outcome variability while generating information that can guide future choices. Adaptive decision-making depends on how individuals balance these competing policies over time (Addicott et al., 2017; Berger-Tal et al., 2014; Cohen et al., 2007; Daw et al., 2006; Melhorn et al., 2015).

An emerging literature has identified factors that shape the explore-exploit trade-off. Short temporal horizons (i.e., limited sampling opportunities), cognitive load, and time pressure all reliably attenuate exploration by reducing the expected value of information, biasing choice selection toward the exploitation of existing knowledge (Daw et al., 2006; Otto et al., 2013; Wilson et al., 2014; Wu et al., 2022). Conversely, when cognitive demands are low and learning opportunities abundant, the value of information increases, promoting exploration. Although these findings highlight the importance of task constraints and cognitive capacity, they largely overlook a fundamental and potentially important feature of everyday decision-making: self-relevance (Epley & Gilovich, 2016; Kunda, 1990; Melhorn et al., 2015). In many contexts, choices have meaningful (e.g., goal-relevant, reputational, emotional) implications for decision makers, raising the possibility that personal relevance may influence how the explore-exploit trade-off is resolved. Critically, however, existing research has not directly examined whether or how self-relevance impacts policy-level arbitration in this respect. A candidate process through which such influence may be exerted is motivated cognition (Kunda, 1990).

## Motivated decision-making

Across perception, attention, and memory, self-related information is prioritized and processed more efficiently than information pertaining to others (Conway, 2005; Golubickis & Macrae, 2023; Sui & Humphreys, 2015, 2017; Symons & Johnson, 1997). These advantages, moreover, extend even to arbitrarily assigned items in inconsequential task settings, indicating that self-relevance exerts a basic influence on social cognition (Golubickis et al., 2018; Sui et al., 2012; Woźniak & Knoblich, 2019). Beyond enhancing processing efficiency, however, self-relevance also biases stimulus evaluation in line with motivational goals. In particular, people are sensitive to the positivity (or otherwise) of behavioral outcomes (Alicke & Sedikides, 2009; Sedikides, 2021; Sedikides & Strube, 1997; Shepperd et al., 2008). Underpinning these effects is approach-avoidance motivation, whereby individuals exhibit a tendency to approach positive outcomes and avoid negative ones, particularly when such experiences have direct consequences for the self (Carver, 2006; Higgins, 1998). Together, these findings suggest that self-relevance not only prioritizes information processing but also biases how outcomes are evaluated and acted upon (Kunda, 1990).

Despite extensive evidence of self-related differences in cognition and social judgment (Mezulis et al., 2004; Polman & Wu, 2020; Shepperd et al., 2008; Sui & Humphreys, 2015; Symons & Johnson, 1997), relatively little work has examined whether comparable biases emerge in the decision policies that support the explore-exploit trade-off. Using associative learning paradigms in conjunction with computational modeling, research has revealed that self-relevance modulates learning rates, response speed, and reward sensitivity (Golubickis & Macrae, 2022; Jalalian et al., 2024; Liao et al., 2021; Lockwood et al., 2022). Notably, self-relevant decisions are associated with increased sensitivity to reward, suggesting that individuals may favor previously reinforced options over uncertain alternatives (Golubickis & Macrae, 2022). However, as prior research has focused primarily on learning rates, it remains unclear whether self-relevance influences the decision policies underpinning the explore-exploit dilemma as people navigate uncertainty.

From a computational perspective, this question can be framed in terms of how self-relevance affects the relative weighting of expected reward and information value; that is, the extent to which choices prioritize immediate payoff versus uncertainty reduction (Addicott et al., 2017; Cohen et al., 2007; Daw et al., 2006; Gershman, 2018; Sutton & Barto, 1998). Computationally, this corresponds to parameters governing uncertainty-directed exploration (e.g., exploration bonuses) and choice stochasticity (e.g., inverse temperature). If self-relevance amplifies the subjective value of positive outcomes (Alicke & Sedikides, 2009), decision makers may place greater weight on reward maximization and discount uncertainty reduction. Such a shift would bias behavior toward exploitation in self-relevant decisions. In contrast, when outcomes relate to others (e.g., friend), reduced motivational investment may permit greater exploration. On this account, self-relevance influences not only outcome valuation but also how decision makers arbitrate between exploration and exploitation.

## The current research

To explore this issue, we employed a restless multi-armed bandit (MAB) task, an established paradigm for examining the explore-exploit trade-off (Bechara et al., 2005; Cohen et al., 2007; Daw et al., 2006; Melhorn et al., 2015; Speekenbrink, 2022; Steyvers et al., 2009). In this task, individuals repeatedly choose between two or more options, each associated with probabilistic rewards (e.g., points) that are initially unknown. Selecting an option yields feedback, allowing the associated values to be learned over time (Addicott et al., 2013; Daw et al., 2006; Speekenbrink & Konstantinidis, 2015). In this way, the task captures the explore-exploit trade-off as decision makers must balance exploiting options that have previously yielded rewards with exploring uncertain alternatives that may produce better outcomes (Addicott et al., 2017; Daw et al., 2006). To probe potential self-other differences in decision-making, participants completed the bandit task either for themselves or on behalf of their best friend (Golubickis & Macrae, 2022; Jalalian et al., 2024). We predicted that self-relevance would increase the weighting of immediate reward relative to information gain, thereby biasing decisions toward exploitation.

## Experiment 1: gains

### Method

#### Participants and design

Two hundred participants (117 females, 83 males; *M* = 25.86, *SD* = 3.27) took part in the experiment. Data were collected through the online platform Prolific Academic (www.prolific.co), with each participant compensated at a rate of £10 per hour. Before the experiment commenced, informed consent was obtained from participants. The protocol was reviewed and approved by the Ethics Committee at the School of Psychology, University of Aberdeen, and the experiment had a 2 (Condition: self vs. friend) between-participants design. To detect a medium-sized effect (*d* = 0.50; Golubickis et al., 2022) with > 80% power, an a priori power analysis conducted in PANGEA (v0.2) indicated a required sample size of 70 participants per condition. Additional participants were recruited to account for potential exclusions due to data quality issues in online testing.

#### Stimulus materials and procedure

**Fig. 1:**
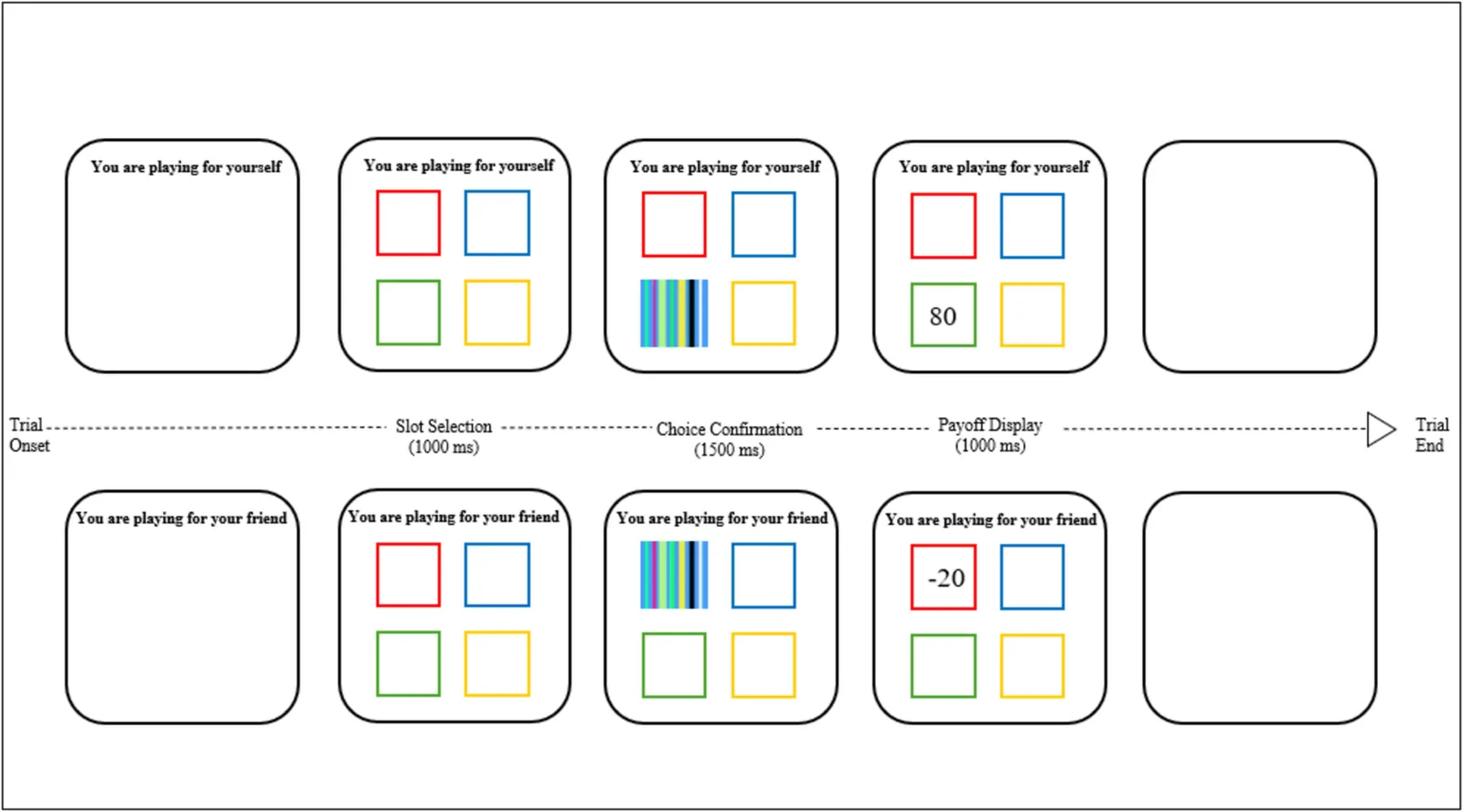
Example of an experimental trial. Upper Panel – Experiment 1 (Gains); Lower Panel – Experiment 2 (Losses)

Participants accessed the computerized task via an Inquisit Web link, where they provided informed consent and demographic information. They were then randomly assigned to either the self-condition or friend-condition. In the self-condition, participants were instructed to perform the task to maximize their own outcomes, whereas in the friend-condition they were directed to maximize outcomes for their best friend. To reinforce these instructions, each trial displayed a reminder (i.e., “You are playing for yourself” or “You are playing for your friend”) at the top of the screen. Participants completed a restless four-armed bandit task (Chakroun et al., 2020; Daw et al., 2006), selecting among four colored squares (i.e., red, green, blue, yellow; see Fig. 1, upper panel), with the goal of maximizing cumulative rewards for the designated target (i.e., self or friend). The squares represented independent bandits and participants were informed that each bandit had a different average payoff, with payoff distributions potentially changing over time (i.e., the task environment was dynamic).

On each trial, the payoff associated with the options was sampled from a Gaussian distribution (*SD* = 4), centered on a time-varying mean, rounded to the nearest integer and constrained to the range 1-100. The expected value of each option evolved across trials according to a decaying Gaussian random walk. Initial means were set to 20, 40, 60, and 80, respectively, and were updated on each trial following the Gaussian diffusion process described by Daw et al. (2006). The minimum possible payoff was 1 and the maximum was 100. Participants completed five practice trials prior to the main task. Each trial began with a blank screen, followed by the appearance of the four squares (i.e., bandits). Participants had 1.5 s to select an option. Failure to respond was recorded as a missed trial and followed by a 1 s warning message. Upon selection, the chosen square was highlighted for 1 s via a flashing grid animation, after which the obtained payoff was displayed for 1 s. The task comprised a single block of 100 trials. At the conclusion of the experiment, participants were shown the total number of points that had been accumulated, debriefed, and thanked.

### Results and discussion

Twenty-one participants were excluded based on predefined criteria: having more than 30% of trials removed due to missing responses, response times faster than 200 ms, or selecting the same option on every trial (Wilson & Collins, 2019). For the remaining participants, trials with latencies less than 200 ms or no responses were removed, resulting in the exclusion of approximately 5% of the data. To examine choice probabilities (i.e., proportion of trials in which a particular option was selected), gains (i.e., points accumulated per trial), and optimal choices (i.e., proportion of trials in which participants selected the option with the highest payoff), a logistic mixed-effects model was adopted with Condition (self vs. friend), Trial Number, and their interaction as fixed effects, and Participants as a random intercept. To capture potential non-linear changes across trials, a model with a spline term for Trial Number and its interaction with Condition was also conducted.

#### Choice probabilities

A likelihood ratio test indicated that the spline model provided a significantly better fit than a linear interaction model, Δχ²(6) = 390.79, *p* < .001. A logistic mixed-effects model including a natural cubic spline for Trial Number revealed a non-linear effect of Trial on choice behavior. All spline terms were statistically significant (|*z*| ≥ 3.46, all *ps* ≤ 0.001). The interaction between Condition and Trial was significant (first spline term: *β* = -0.887, SE = 0.179, *z* =-4.96, *p* < .001), indicating that the effect of Condition (self vs. friend) varied non-linearly across trials. The main effect of Condition (evaluated at the spline reference point) was positive (*β* = 0.346, SE = 0.155, *z* = 2.24, *p* = .025), corresponding to an odds ratio of 1.41.

**Fig. 2:**
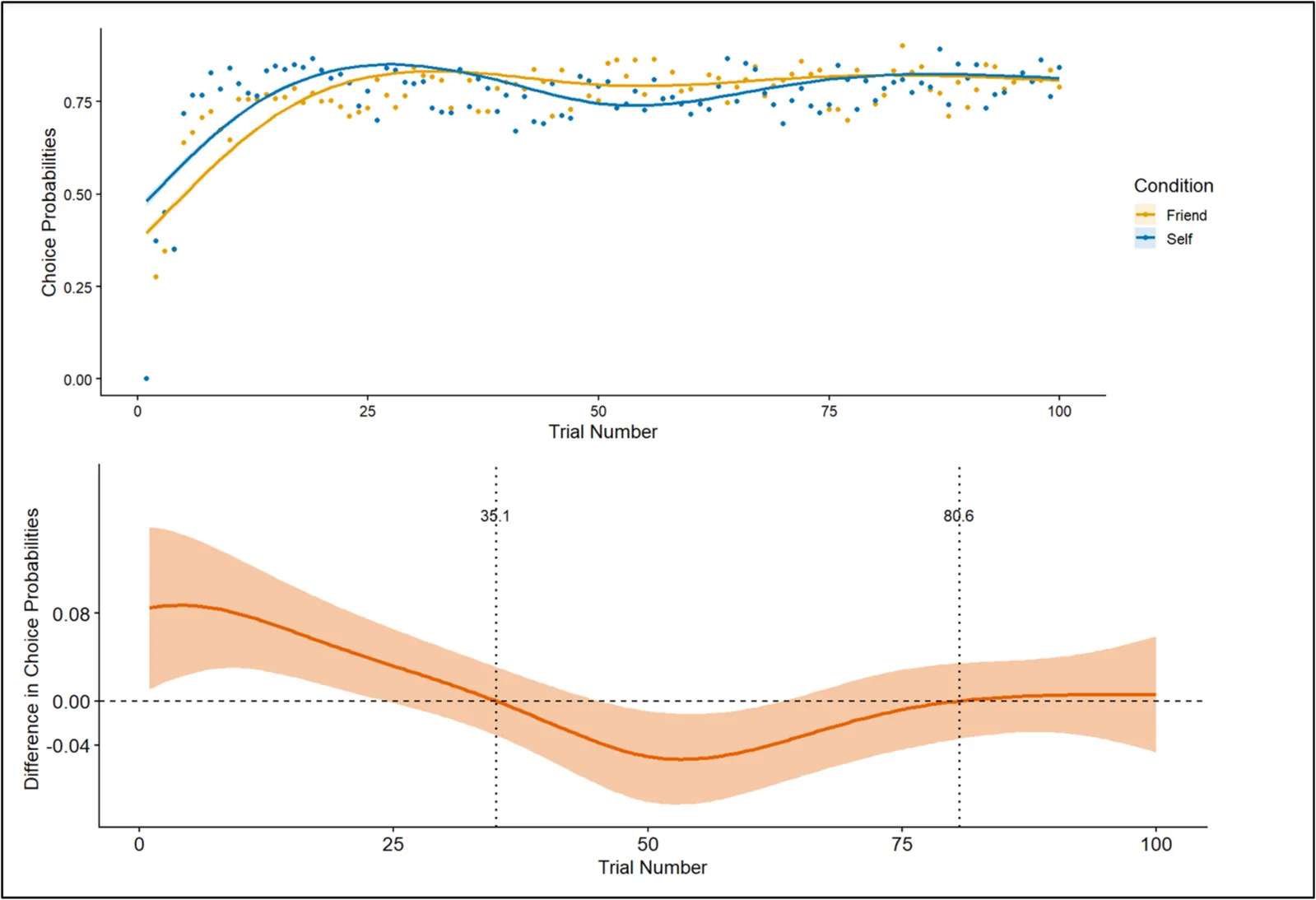
Averaged trial-level choice probabilities are shown as points, with model-predicted lines (Upper Panel) and a spline of the difference in predicted probabilities between conditions (Lower Panel). Shaded regions represent 95% confidence intervals (CIs). Values above zero suggest higher self-exploitation, whereas values below zero suggest higher friend-exploitation. Vertical dotted lines mark crossover points where shifts occurred

Model-implied predicted probabilities showed that the self-friend difference varied dynamically across trials. The estimated difference changed direction over the course of the task, with approximate crossover points around trials 35 and 81. Choice probabilities were higher in the self-condition (peak difference ≈ + 0.08) during the early stages of the task, and higher in the friend-condition (minimum difference ≈ − 0.05) mid-task. Thus, the effect of Condition was not constant but evolved non-linearly over the course of learning (see Fig. 2).

#### Gains

A likelihood ratio test indicated that the spline model provided a significantly better fit than a linear interaction model, Δχ²(6) = 322.97, *p* < .001. A linear mixed-effects model including a natural cubic spline for Trial Number (4 degrees of freedom) indicated a non-linear effect of Trial on gains. Spline terms were statistically significant (|*t*| ≥ 3.93, all *ps* ≤ 0.001), except for the second term (*p* = .06). There was also a significant interaction between Condition and Trial (first spline term: *β* =-6.03, SE = 1.10, *t* = -5.47, *p* < .001; fourth spline term: *β* = -2.09, SE = 1.02, *t* = -2.03, *p* = .042), indicating that the trajectory of gains differed as a function of whether participants were making decisions for themselves or for a friend.

**Fig. 3:**
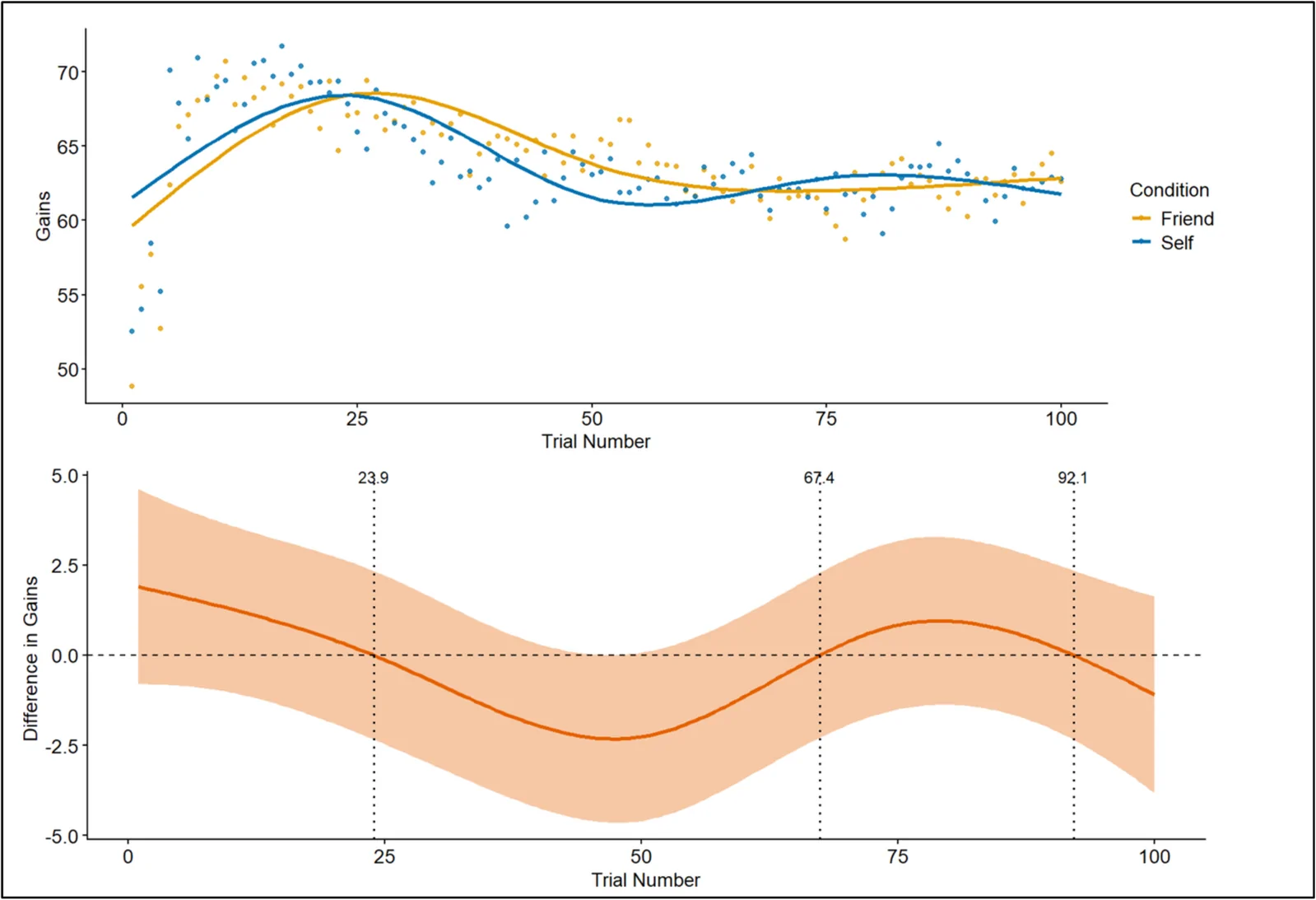
Averaged trial-level gains are shown as points, with model-predicted lines (Upper Panel) and a spline of the difference in predicted gains between conditions (Lower Panel). Shaded regions represent 95% confidence intervals (CIs). Values above zero indicate higher self-gains, whereas values below zero indicate higher friend-gains. Vertical dotted lines mark crossover points where shifts occurred

Model-implied predicted values showed that the self-friend difference varied dynamically across trials. The estimated difference changed direction over the course of the task, with approximate crossover points around trials 24, 67, and 92. Gains were higher in the self-condition (peak difference ≈ + 2) during the early stages of the task, higher in the friend-condition (minimum difference ≈ -2) mid-task, and higher in the self-condition (peak difference ≈ + 1) toward the end of the task. Overall, gains evolved non-linearly across trials (see Fig. 3).

#### Optimal choices

A likelihood ratio test indicated that the spline model provided a significantly better fit than a linear interaction model, Δχ²(6) = 218.16, *p* < .001. A logistic mixed-effects model including a natural cubic spline for Trial Number (4 degrees of freedom) revealed a non-linear effect of Trial on the probability of making the optimal choice (first & third spline terms: |*z*| ≥ 1.78, *ps* ≤ 0.001). There was also a significant interaction between Condition and Trial (first spline term: *β* = -0.64, SE = 0.16, *z* = -3.88, *p* < .001), indicating that the relationship between trial progression and the probability of choosing the optimal arm differed depending on whether participants were making decisions for themselves or for a friend.

**Fig. 4:**
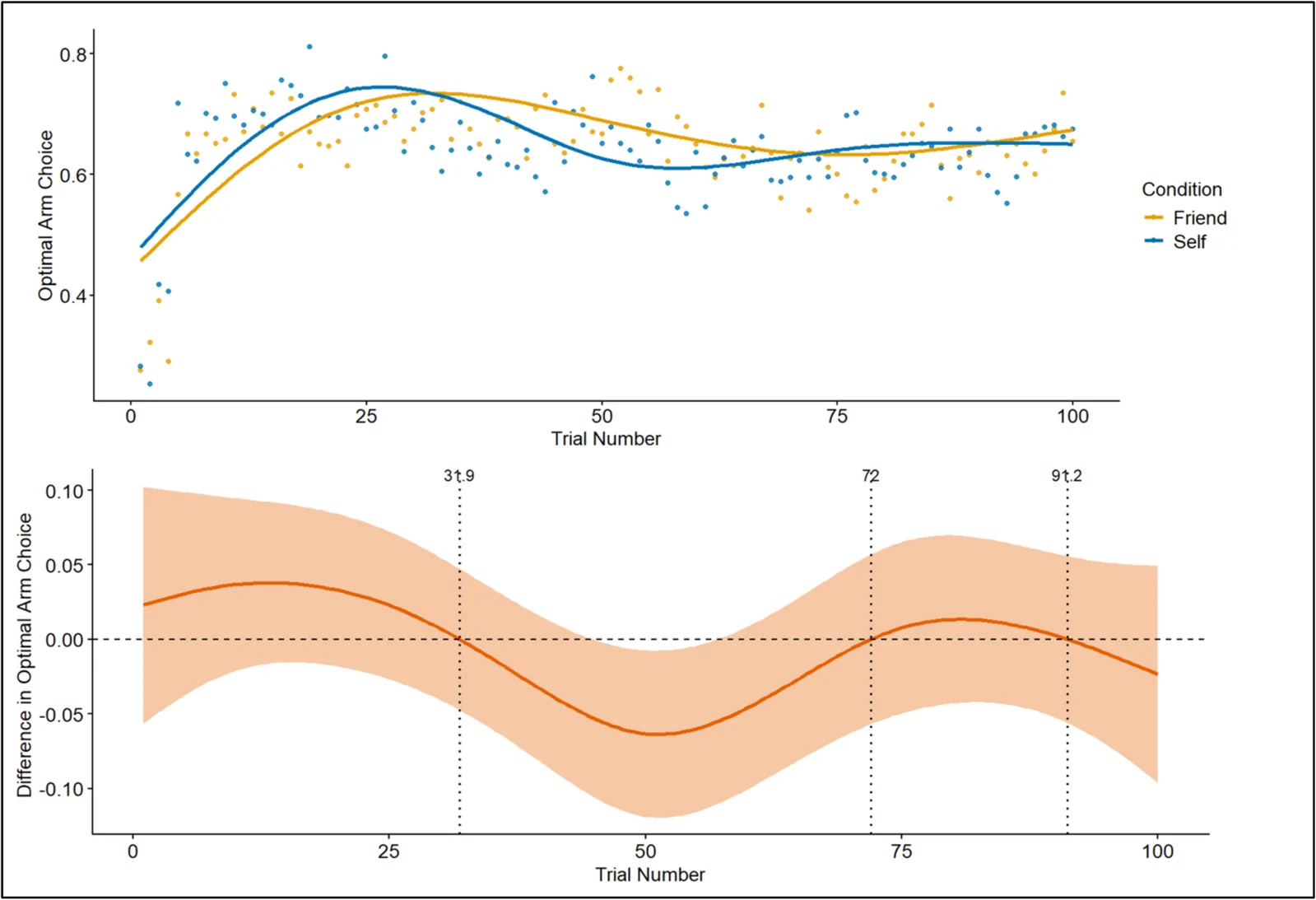
Averaged trial-level optimal choices are shown as points, with model-predicted lines (Upper Panel) and a spline of the difference in predicted optimal arm choice between conditions (Lower Panel). Shaded regions represent 95% confidence intervals (CIs). Values above zero indicate more optimal choices for self, whereas values below zero indicate more optimal choices for friend. Vertical dotted lines mark crossover points where shifts occurred

Model-implied predicted probabilities showed that the self-friend difference varied dynamically across trials. The difference changed direction over the course of the task, with approximate crossover points at around trials 32, 72, and 91. The probability of making the optimal choice was higher in the self-condition (peak difference ≈ + 0.02) during the early stages of the task, higher in the friend-condition (minimum difference ≈ -0.06) mid-task, and higher in the self-condition (peak difference ≈ + 0.01) toward the end of the task. Overall, the probability of making optimal choices varied non-linearly across trials (see Fig. 4).

The results of Experiment 1 indicate that self-relevance systematically modulates how participants allocate choices over the course of learning in a restless MAB task. Across all three outcome measures—choice probabilities, gains, and optimal choices—the effect of Condition varied non-linearly across trials, suggesting that differences between self- and friend-related decision-making were not constant but evolved dynamically over time. Dynamic shifts in choice probability are consistent with the explore-exploit dilemma, whereby participants must balance trying new options against leveraging known rewards (Cohen et al., 2007; Sutton & Barto, 1998). Early in learning, participants showed enhanced selection of previously successful options when deciding for themselves (vs. friend), consistent with the rapid exploitation of immediate rewards. This tendency attenuated mid-task, where increased variability in self-related choices suggests a shift toward exploration, potentially reflecting uncertainty-driven re-sampling of alternatives. Later in the task, self-related choices converged toward renewed exploitation, consistent with the acquisition of stabilized value estimates. Together, these findings suggest that self-relevance influences not only choice consistency but the temporal dynamics of the explore–exploit trade-off.

Experiment 1 examined decision-making in an environment framed around gains. Of additional interest are situations in which decisions are directed toward the opposite goal, the minimization of losses (Chin et al., 2023; Krueger et al., 2017; Lejarraga & Hertwig, 2017; Teodorescu & Erev, 2014). A substantial body of evidence demonstrates that outcome valence exerts a potent influence on decision-making, such that people tend to be risk-averse when pursuing gains but risk-seeking when facing losses (Kahneman & Tversky, 1979; Tversky & Kahneman, 1992). A comparable asymmetry emerges under conditions of uncertainty, with individuals exhibiting ambiguity-aversion for gains but ambiguity-seeking for losses (Di Mauro & Maffioletti, 1996; Du & Budescu, 2005; Ho et al., 2001; Kahn & Sarin, 1988). In the current context, this suggests that losses affecting the self (vs. friend) may carry heightened subjective significance, thereby increasing motivation to avoid negative outcomes. As such, self-relevance may amplify uncertainty-seeking under losses, insofar as exploratory sampling reduces the risk of accruing larger penalties. Experiment 2 examined this prediction using a restless MAB task in which outcomes were framed as losses.

## Experiment 2: losses

### Method

#### Participants and design

One hundred and seventy participants (88 females, 82 males; *M* = 25.90, *SD* = 3.24) took part in the experiment. Data were collected through the online platform Prolific Academic (www.prolific.co), with each participant compensated at a rate of £10 per hour. Before the experiment commenced, informed consent was obtained from participants. The protocol was reviewed and approved by the Ethics Committee at the School of Psychology, University of Aberdeen, and the experiment had a 2 (Condition: self vs. friend) between-participants design. The power analysis followed the same procedure and parameters as in Experiment 1.

#### Stimulus materials and procedure

The procedure was as in Experiment 1, but with a single modification. On this occasion, participants’ choices incurred losses (see Fig. 1, lower panel). In the self-condition, participants were instructed to perform the task to minimize their own losses, whereas in the friend-condition they were directed to minimize losses for their best friend. On each trial, the outcome associated with each option was sampled from a Gaussian distribution (*SD* = 4), centered on a time-varying mean, rounded to the nearest integer and constrained to the range 1-100. The expected value of each option evolved across trials according to a decaying Gaussian random walk. Initial means were set to -20, -40, -60, and − 80, respectively, and were updated on each trial (Daw et al., 2006). The task adopted a pure loss frame in which participants began at zero and accumulated only penalties (Krueger et al., 2017). This design draws on prospect theory (Kahneman & Tversky, 1979) and the literature on decisions from experience (Barron & Erev, 2003; Hertwig et al., 2004), allowing the examination of exploration-exploitation under sustained loss incentives. As in Experiment 1, the task comprised a single block of 100 trials. At the conclusion of the experiment, participants were shown the total number of points that had been lost, debriefed, and thanked.

### Results and discussion

Twenty-seven participants were excluded based on the same predefined criteria as in Experiment 1. For the remaining participants, trials with response latencies below 200 ms or missing responses were removed, resulting in the exclusion of approximately 9% of the data. The analytical strategy was identical to that used in Experiment 1.

#### Choice probabilities

**Fig. 5:**
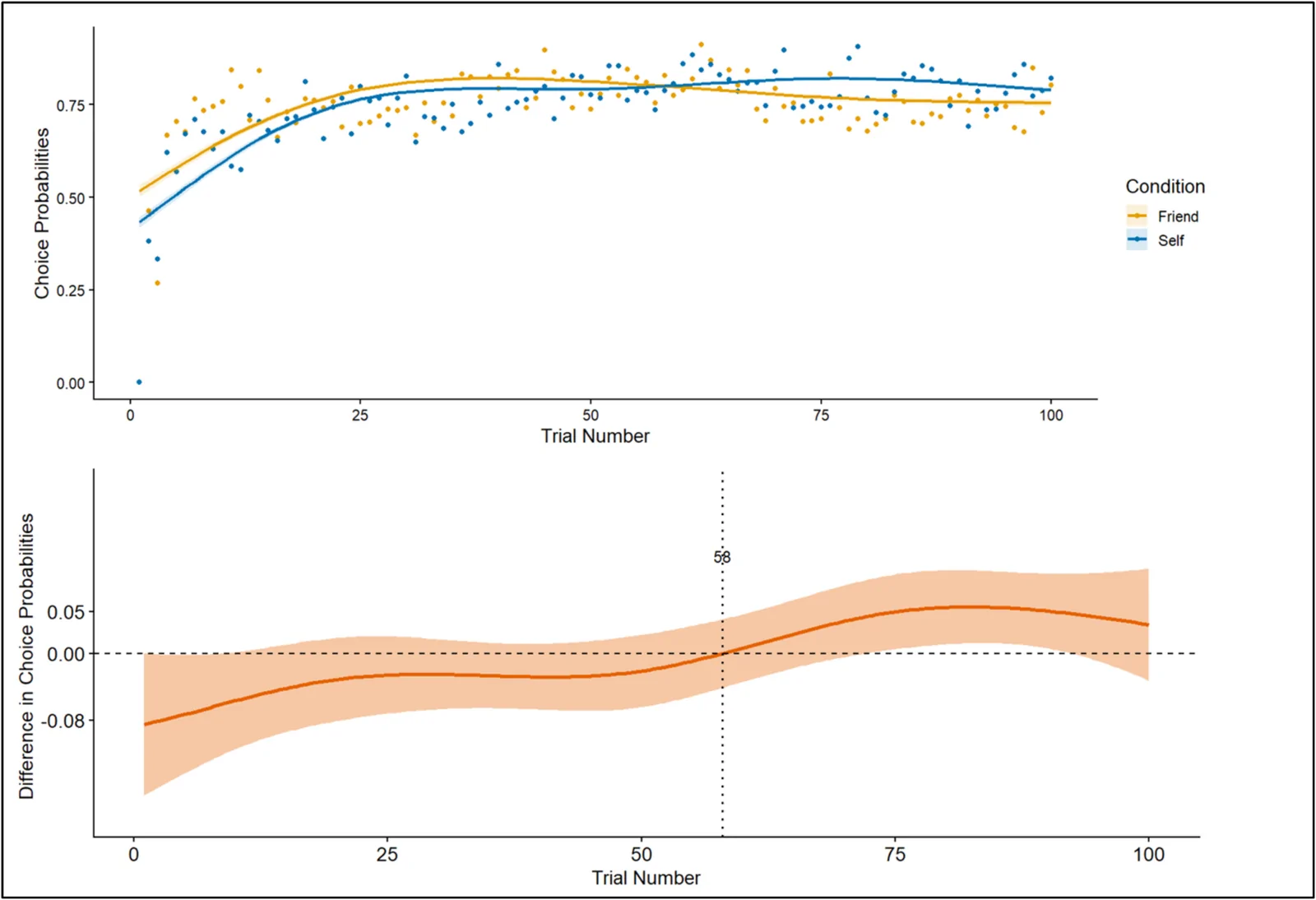
Averaged trial-level choice probabilities are shown as points, with model-predicted lines (Upper Panel) and a spline of the difference in predicted probabilities between conditions (Lower Panel). Shaded regions represent 95% confidence intervals (CIs). Values below zero suggest higher self-exploration, whereas values above zero indicate higher friend-exploration. Vertical dotted lines mark crossover points where shifts occurred

A likelihood ratio test indicated that the spline model provided a significantly better fit than a linear interaction model, Δχ²(6) = 223.3, *p* < .001. A linear mixed-effects model including a natural cubic spline for Trial Number (4 degrees of freedom) revealed a non-linear effect of Trial on choice behavior (spline terms: |*z*| ≥ 4.45, all *ps* ≤ 0.001, except the fourth term, *p* = .177). There was evidence that the effect of Condition varied over the course of the task. Although the main effect of Condition at the spline reference point failed to reach significance (*β* =-0.341, SE = 0.175, z = -1.94, p = .052; OR = 0.71), several spline interaction components were significant (second spline term: β = 0.754, SE = 0.209, z = 3.61, p < .001; third spline term: β = 0.868, SE = 0.383, z = 2.27, p = .023), indicating that the self-friend difference changed non-linearly across trials (Fig. 5).

Model-implied predicted probabilities showed that the self-friend difference varied dynamically across trials. The estimated difference changed direction over the course of the task, with an approximate crossover point around trial 58. Choice probabilities were lower in the self-condition (maximum difference ≈ − 0.08) during the early stages of the task, then higher in the self-condition (peak difference ≈ + 0.05) for the remainder of the task (see Fig. 5).

#### Losses

A likelihood ratio test indicated that the spline model provided a significantly better fit than a linear interaction model, Δχ²(6) = 226.6, *p* < .001. A linear mixed-effects model including a natural cubic spline for Trial Number (4 degrees of freedom) revealed a non-linear effect of Trial on losses (spline terms: |*t*| ≥ -3.93, all *ps* ≤ 0.001, except the second term, *p* = .06). There was also a significant interaction between Condition and Trial (first spline term: *β* = -6.03, SE = 1.10, *t* = -5.47, *p* < .001; and fourth spline term: *β* = -2.09, SE = 1.02, *t* = -2.03, *p* = .042), indicating that the trajectory of losses differed as a function of whether participants were making decisions for themselves or for a friend.

**Fig. 6:**
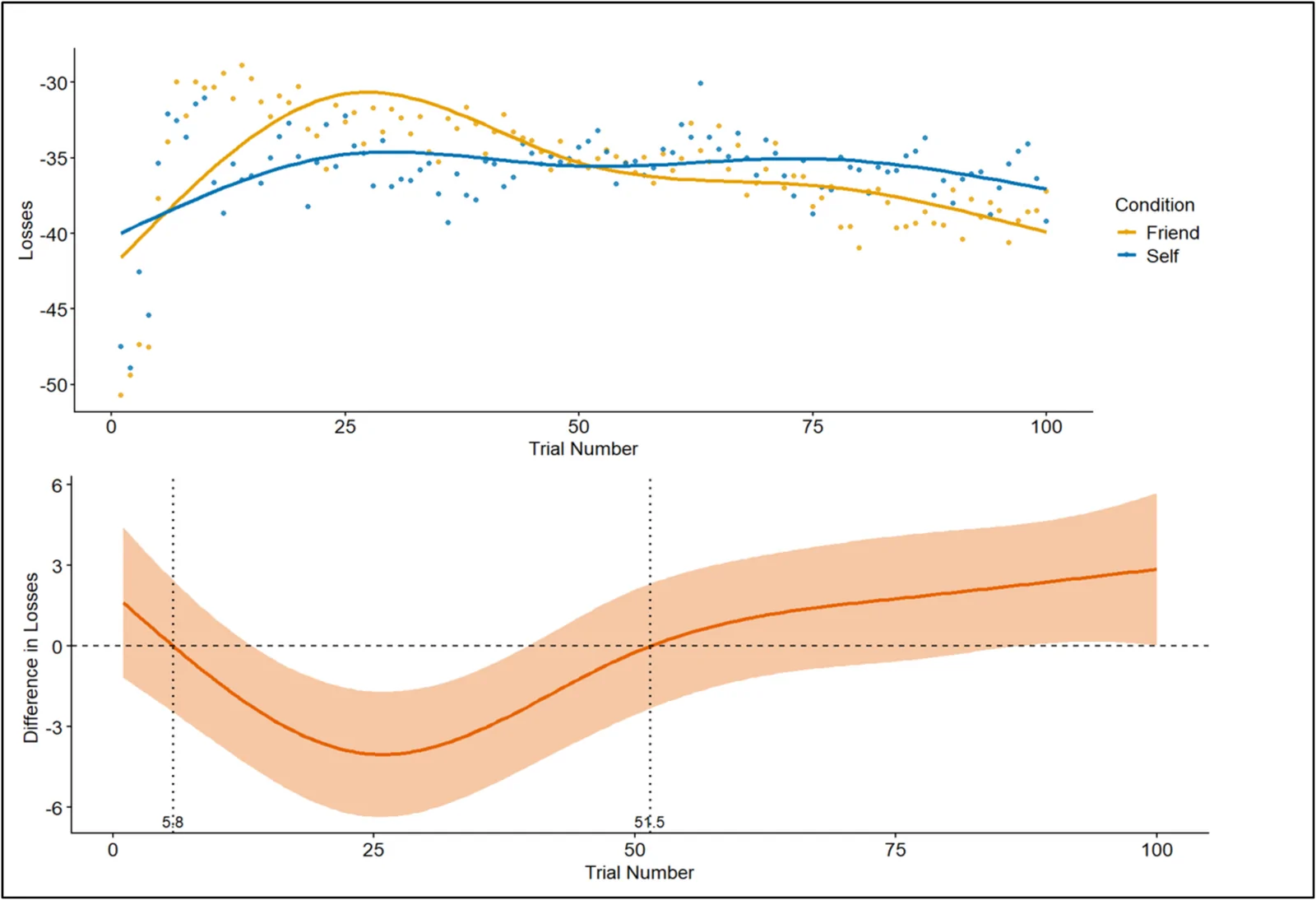
Averaged trial-level losses are shown as points, with model-predicted lines (Upper Panel). A spline of the difference in predicted losses between conditions. Shaded regions represent 95% confidence intervals (CIs). Values above zero indicate higher self-losses, whereas values below zero indicate higher friend-losses. Vertical dotted lines mark crossover points where this advantage reverses (Lower Panel)

Model-implied predicted losses showed that the self-friend difference varied dynamically across trials. The estimated difference changed direction over the course of the task, with approximate crossover points around trials 24, 67, and 92. Losses were lower in the self-condition (peak difference ≈ + 2) during the early stages of the task, lower in the friend-condition (minimum difference ≈ -2) mid task, and lower in the self-condition (peak difference ≈ + 1) toward the end of the task. Overall, losses evolved non-linearly across trials (see Fig. 6).

#### Optimal choices

A likelihood ratio test indicated that the spline model provided a significantly better fit than a linear interaction model, Δχ²(6) = 116.5, *p* < .001. A logistic mixed-effects model with a natural cubic spline for Trial Number (4 degrees of freedom) revealed a non-linear effect of Trial on optimal choices. All spline terms were statistically significant (|*z*| ≥ -3.22, *ps* ≤ 0.05). There was also a significant interaction between Condition and Trial (first spline term: *β* = 0.40, *SE* = 0.19, *z* = 2.15, *p* < .03; fourth spline term: *β* = 0.77, *SE* = 0.18, *z* = 4.37, *p* < .001) indicating that the relationship between trial progression and the probability of choosing the optimal arm differed depending on whether participants were making decisions for themselves or for a friend

**Fig. 7:**
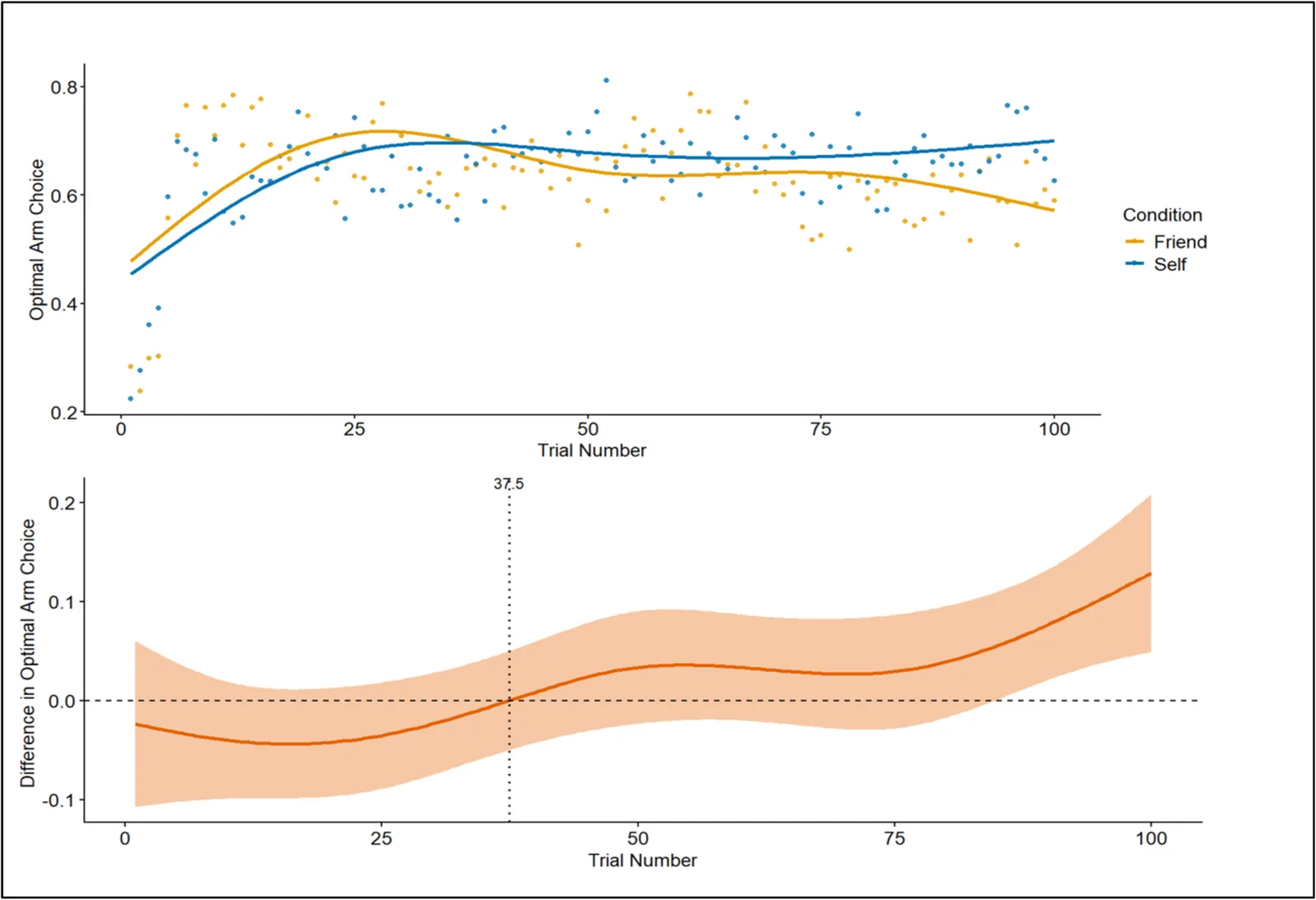
Averaged trial-level optimal choices are shown as points, with model-predicted lines (Upper Panel) and a spline of the difference in predicted optimal arm choice between conditions (Lower Panel). Shaded regions represent 95% confidence intervals (CIs). Values above zero indicate more optimal choices for self, whereas values below zero indicate more optimal choices for friend. Vertical dotted lines mark crossover points where shifts occurred

Model-implied predicted probabilities showed that the self-friend difference varied dynamically across trials. The difference changed direction over the course of the task, with approximate crossover points at around trials 6 and 52. The probability of making the optimal choice was higher in the friend-condition (minimum difference ≈ − 0.05) during the early stages of the task, and higher in the self-condition (peak difference ≈ + 0.1) later in the task. Overall, the probability of making optimal choices varied non-linearly across trials (see Fig. 7).

In a loss-framed environment, the results of Experiment 2 reveal that self-relevance shapes how participants allocate choices in a restless MAB task (Chakroun et al., 2020; Daw et al., 2006). On all three outcome measures (i.e., choice probabilities, losses, and optimal choices), the effect of Condition varied non-linearly across trials, indicating dynamic differences between self- and friend-related decisions. Early in the task, participants exhibited greater exploration and reduced optimality when choosing for themselves, consistent with increased uncertainty-seeking when personal outcomes are at stake. In contrast, decisions made for a friend were initially more conservative and closely aligned with loss minimization. As learning progressed and uncertainty diminished this pattern reversed, such that self-directed choices became increasingly optimal, reflected in higher selection of advantageous outcomes and reduced losses. This shift suggests a transition from early uncertainty-driven exploration to later exploitation of learned value. Collectively, these findings point to a temporally evolving balance between exploration and exploitation, with self-relevant decisions becoming increasingly optimized as learning unfolds.

## Computational analysis

The behavioral effects observed across both experiments—varying only in whether gains or losses were under investigation—suggest that self-relevance modulates how participants transition between exploration and exploitation over time. From a computational perspective, such dynamics can arise from changes in value updating (i.e., learning rate), the stochasticity of choice (i.e., inverse temperature), or explicit exploration mechanisms. Accordingly, to identify the latent cognitive processes underlying self-friend differences in decision-making, we fitted a series of hierarchical reinforcement learning models to trial-by-trial choice data. The models combined a delta-rule learning algorithm with a softmax choice function, providing a principled framework for dissociating value updating from decision policy and for quantifying the explore-exploit trade-off in probabilistic environments (Daw et al., 2006; Sutton & Barto, 1998). Specifically, on each trial, expected values (Q-values) were updated according to a prediction-error rule. Choices were generated via a softmax function, with the inverse temperature parameter (*β*) governing the extent to which value differences were translated into deterministic choice and thus greater exploitation (Daw et al., 2006).

To capture components of decision-making beyond value-based choice, four computational models were contrasted (Chakroun et al., 2020). In each model, the delta learning rule was combined with four different choice rules. Model 1 employed a conventional softmax choice rule with a single inverse temperature parameter (*β*), representing the degree of random exploration. Model 2 added an additional free parameter (*φ*) that reflected an exploration bonus which increased the attractiveness of options with greater estimated uncertainty, thereby capturing directed exploration. Model 3 included an additional free parameter (*ρ*) capturing a perseveration bonus that modeled the tendency to repeat the option selected on the previous trial (i.e., sticky choice). Finally, Model 4 added a parameter (𝛾) that indexed random exploration as a function of the overall uncertainty across all bandits. Model parameters were estimated hierarchically using Bayesian methods, with individual-level estimates constrained by group-level prior distributions. To assess the influence of social context and outcome valence, parameters were estimated separately for each combination of the 2 (Condition: self vs. friend) X 2 (Environment: gains vs. losses) factorial design, allowing latent decision-making processes to vary across experimental conditions.

**Fig. 8:**
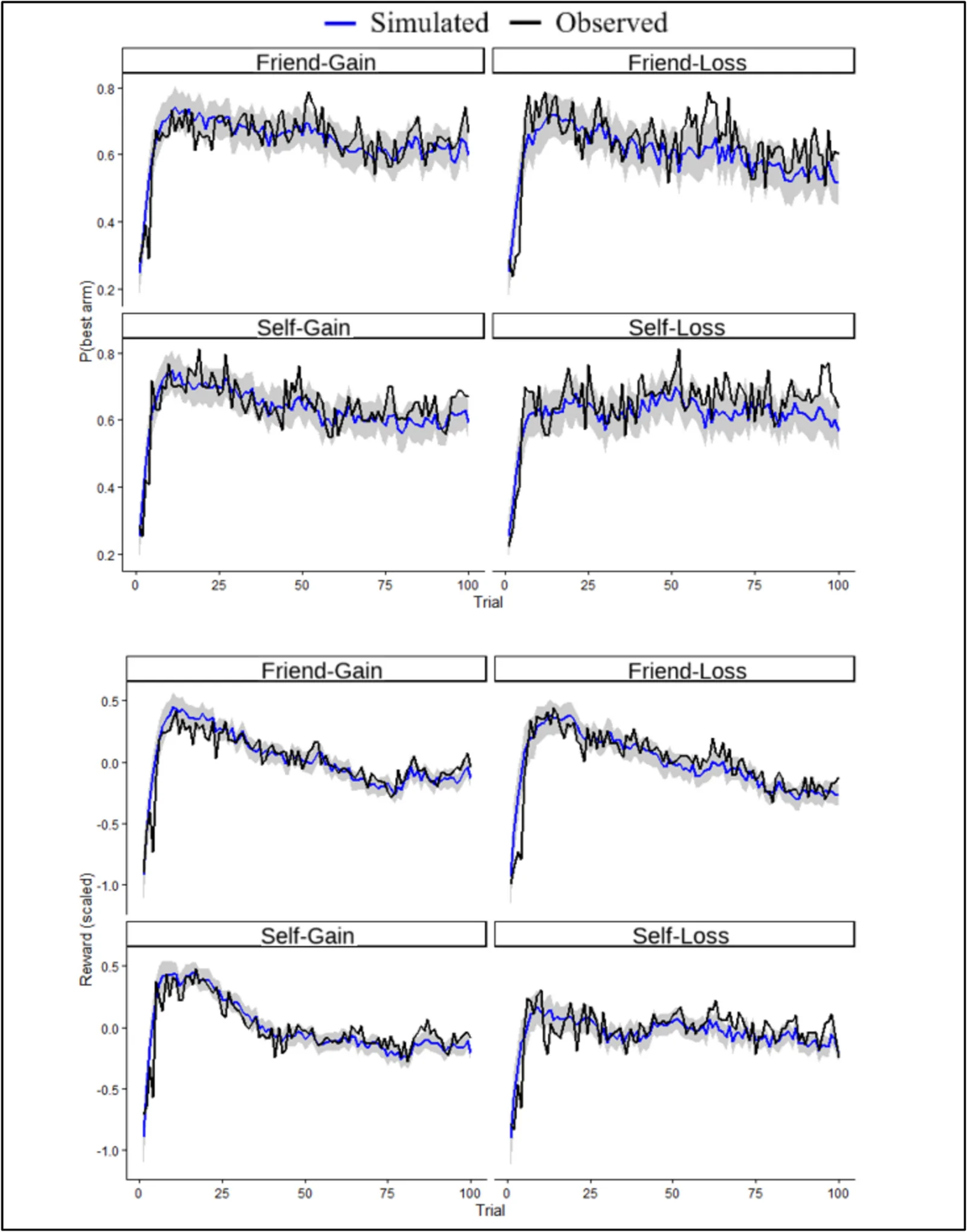
Posterior predictive checks from the best-fitting model, illustrating trial-wise scaled reward and optimal choice probability across conditions

All models were estimated using hierarchical Bayesian methods and compared using approximate leave-one-out cross-validation (LOOCV), a principled measure of out-of-sample predictive accuracy (Vehtari et al., 2017). Model comparison indicated that Model 4 yielded the best fit (Leave-one-out information criterion [LOOIC]: Model 1 ≈ 43411, Model 2 ≈ 41813, Model 3 ≈ 39233, Model 4 ≈ 39149). Unlike traditional goodness-of-fit measures, cross-validation methods such as LOOCV do not systematically favor more complex models. As such, they do not require an additional penalty term to guard against overfitting. The modeling analysis indicated that behavior was best explained by a combination of value learning, exploration processes, and action persistence, consistent with prior work showing that multiple computational mechanisms jointly underpin the explore-exploit trade-off. Posterior predictive checks confirmed that Model 4 captured key behavioral effects, including condition-specific learning trajectories and choice probabilities (see Fig. 8).

Parameter estimates from the best-fitting model provided a detailed account of how Condition and Environment shaped distinct components of the decision-making process.

### Learning rate (α)

**Fig. 9:**
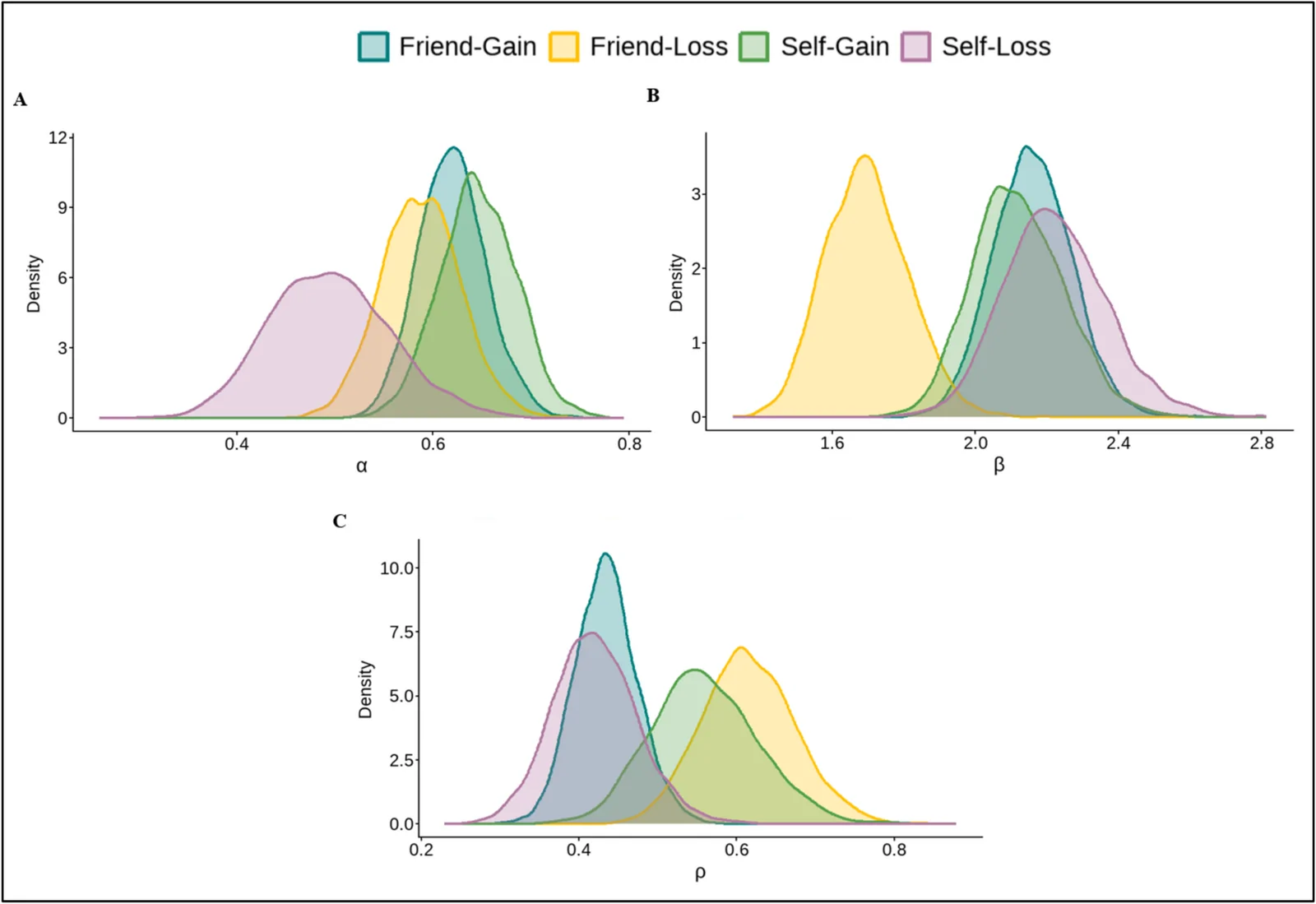
Posterior distributions of model parameters from the best-fitting model, showing Condition X Environment interactions on Panel A: learning rate (α), Panel B: inverse temperature (β), and Panel C: perseveration (ρ)

There was very strong evidence (see Lee & Wagenmakers, 2013) for a main effect of Environment (pBayes(gains > losses) = 0.026, ΔM = 0.091, BF₁₀ = 37.46) and anecdotal evidence for a main effect of Condition (pBayes(friend > self) = 0.228, ΔM = 0.033, BF₁₀ = 3.38). These effects were qualified, however, by an Environment X Condition interaction (pBayes = 0.09, ΔM = 0.119, BF₁₀ = 10; see Fig. 9). Follow-up contrasts indicated anecdotal evidence that learning rates were higher for self than friend for gains (pBayes(self > friend) = 0.299, ΔM = 0.026, BF₁₀ = 2.34), and moderate evidence that learning rates were higher for friend than self for losses (pBayes(friend > self) = 0.097, ΔM = 0.093, BF₁₀ = 9.31). Within Condition, there was strong evidence for a higher learning rate in gains relative to losses for self (pBayes(gains > losses) = 0.018, ΔM = 0.151, BF₁₀ = 54.56), but only anecdotal evidence for this difference for friend (pBayes(gains > losses) = 0.268, ΔM = 0.032, BF₁₀ = 2.73). These findings indicate that learning was primarily driven by outcome valence (i.e., gains faster than losses), but this effect was more pronounced for self than friend.

### Inverse temperature (β)

There was strong evidence for a main effect of Environment (pBayes(gains > losses) = 0.07, ΔM = 0.180, BF₁₀ = 13.29) and very strong evidence for a main effect of Condition (pBayes(self > friend) = 0.028, ΔM = 0.245, BF₁₀ = 34.71). Critically, however, these effects were qualified by an Environment X Condition interaction (pBayes = 0.011, ΔM = 0.566, BF₁₀ = 89.91; see Fig. 9). Decomposition of the interaction revealed anecdotal evidence for a difference between self and friend for gains (pBayes(self > friend) = 0.402, ΔM = 0.039, BF₁₀ = 1.48), but extremely strong evidence for higher inverse temperature for self than friend for losses (pBayes(self > friend) = 0.003, ΔM = 0.527, BF₁₀ = 332.33). Within condition, there was anecdotal evidence of a gain-loss difference for self (pBayes(losses > gains) = 0.296, ΔM = 0.103, BF₁₀ = 2.38), but extremely strong evidence for this difference for friend (pBayes(gains > losses) = 0.002, ΔM = 0.464, BF₁₀ = 499). Taken together, these findings suggest that decision policies for the self were comparatively stable and consistently exploitative across gain and loss environments. In contrast, decision-making for a friend was more context dependent.

### Directed exploration bonus (φ)

There was strong evidence for a main effect of Environment (*p*_*Bayes*_(gains > losses) = 0.017, ΔM = 0.0073, BF₁₀ = 57.82), but only anecdotal evidence for both a main effect of Condition (*p*_*Bayes*_(friend > self) = 0.383, ΔM = 0.0010, BF₁₀ = 1.61) and an Environment X Condition interaction (*p*_*Bayes*_ = 0.360, ΔM = 0.0026, BF₁₀ = 1.78). This suggests that strategic exploration driven by uncertainty was primarily determined by the task environment, with no reliable modulation by condition.

### Perseveration (ρ)

There was no evidence for main effects of either Condition (pBayes(friend > self) = 0.264, ΔM = 0.035, BF₁₀ = 2.79) or Environment (pBayes(losses > gains) = 0.352, ΔM = 0.021, BF₁₀ = 1.84). However, there was extremely strong evidence for a Condition X Environment interaction (pBayes < 0.001, ΔM = 0.320, BF₁₀ > 999; see Fig. 9), indicating that choice repetition was selectively expressed in specific condition-environment combinations. Follow-up contrasts revealed strong evidence for higher perseveration for self than friend in gains (pBayes(self > friend) = 0.05, ΔM = 0.125, BF₁₀ = 19), and very strong evidence for lower perseveration for self than friend in losses (pBayes(friend > self) = 0.007, ΔM = 0.195, BF₁₀ =141.86). Within condition, there was also strong evidence of higher perseveration in gains than losses for self (pBayes(gains > losses) = 0.053, ΔM = 0.139, BF₁₀ = 17.87), and extremely strong evidence of lower perseveration in gains than losses for friend (pBayes(losses > gains) = 0.002, ΔM = 0.181, BF₁₀ = 499). These findings indicate that perseverative behavior was not governed by a general bias to repeat prior choices but instead was highly context dependent. Specifically, participants showed adaptive flexibility when making self-relevant decisions—persisting after gains but disengaging after losses—whereas decisions made on behalf of others exhibited the opposite tendency, with increased repetition following losses.

### Random exploration (γ)

There was strong evidence for a main effect of Environment (p_Bayes_(losses > gains) = 0.017, ΔM = 1.219, BF₁₀ = 57.82), and moderate evidence for both a main effect of Condition (p_Bayes_(losses > gains) = 0.122, ΔM = 0.678, BF₁₀ = 7.2) and an Environment X Condition interaction (p_Bayes_ = 0.125, ΔM = 1.363, BF₁₀ = 7). These results indicate that random exploration was modulated by outcome valence, but largely insensitive to condition.

## General discussion

Across two experiments and a combined computational modeling analysis, the current research examined how self-relevance influences the resolution of the exploration-exploitation trade-off. Extending reinforcement-learning accounts of adaptive behavior (e.g., Cohen et al., 2007; Sutton & Barto, 1998), we tested whether decisions made for oneself versus another (i.e., best friend) systematically alter the balance between reward maximization and information seeking. The findings provided evidence that self-relevance exerts a reliable and temporally dynamic influence on decision-making, with potential implications for how motivational factors interact with core learning and choice mechanisms.

At the behavioral level, self-other differences were not static but evolved non-linearly over time, shaping when participants transitioned between exploration and exploitation. In the context of gains (i.e., Experiment 1), participants initially exhibited greater exploitation when choosing for themselves, consistent with prioritizing immediate reward (Daw et al., 2006). This early advantage gave way to a mid-task shift toward increased exploration, before returning to exploitation as stable value estimates were acquired (Auer et al., 2002; Sutton & Barto, 2018). In the context of losses (Experiment 2), a complementary pattern emerged, such that participants were initially more exploratory when making self-relevant decisions—consistent with uncertainty-seeking under potential loss (Kahneman & Tversky, 1979)—but gradually shifted toward more optimal, exploitative responding as learning progressed. Across both experiments, self-relevance amplified sensitivity to the motivational properties of the environment, producing early-stage biases in exploration or exploitation that were recalibrated as uncertainty diminished (Gershman, 2018; Niv et al., 2015). These findings indicate that self-relevance influences the temporal dynamics of decision-making rather than inducing a fixed bias toward exploration or exploitation.

An additional computational modeling analysis indicated that self-relevance moderated how participants learned from and acted on outcomes. Learning for oneself was more strongly driven by positive outcomes, with faster updating following gains than losses, an asymmetry that was weaker when learning for a friend. Decision-making for the self was also more stable and consistently exploitative across contexts (Daw et al., 2006; Gershman, 2018; Sutton & Barto, 2018), while choices for a friend were more sensitive to whether outcomes were gains or losses. In terms of behavior, participants showed a flexible pattern when acting for themselves—tending to stick with choices after gains but shift after losses—whereas decisions for a friend were less adaptive, with a greater tendency to persist following negative outcomes. Overall, these findings suggest that self-relevance promotes more consistent, reward-sensitive, and adaptively flexible decision-making compared to decisions made on behalf of a friend. In this way, the results support computational accounts that distinguish between value learning and action selection (Dayan & Daw, 2008; Gershman & Schoenbaum, 2017) and identify decision policy as a primary locus of self-referential influence.

### Theoretical implications

The current findings suggest an interesting application of self-enhancement theory to the domain of value-based decision-making under uncertainty, whereby motivational biases associated with self-relevance influence how individuals resolve the exploration-exploitation dilemma. To date, classic accounts of self-enhancement have emphasized preferential evaluation, selective information processing, and asymmetric attributional styles as strategies through which a positive self-concept can be bolstered or protected (Alicke & Sedikides, 2009; Sedikides, 2021). Of theoretical note, the current results suggest that these motives may also operate at a more fundamental computational level, influencing not only how outcomes are interpreted but how decision policies are deployed in ambiguous environments. Whether the task context entailed gains or losses, self-relevance was associated with shifts in choice consistency (i.e., inverse temperature) and action repetition (i.e., perseveration). In other words, self-enhancement motives (e.g., bolster-self and protect-self) were realized through common computational mechanisms. From a social-cognitive perspective, these findings position self-enhancement as a potentially important determinant of adaptive decision-making (Alicke & Sedikides, 2009; Sedikides, 2021; Sedikides & Strube, 1997). Pursuit of a positive self-concept biases not only what individuals believe, but also how they learn from and act upon their decisions.

By demonstrating that self-relevance influenced the computational control of learning and choice (Golubickis & Macrae, 2022; Jalalian et al., 2024; Liao et al., 2021; Lockwood et al., 2022), the current findings advance contemporary accounts of self-function. Whereas dominant frameworks highlight the role of self-relevance in perceptual/attentional processing (Humphreys & Sui, 2016; Sui & Humphreys, 2015, 2017), the current results indicate that personal significance alters the balance between exploration and exploitation during decision-making. Specifically, the temporally dynamic patterns observed across both gain and loss contexts suggest that self-referential processing interacts with uncertainty in a flexible manner, amplifying early exploitation under gains but promoting initial exploration under losses, before converging on optimal policies. These findings are consistent with a heuristic account of self-function (Golubickis et al., 2026), in which the effects of self-relevance emerge flexibly as a function of the decision-maker’s computational capacities and, crucially, the structure of the task environment (Gigerenzer, 2008; Gigerenzer & Gaissmaier, 2011). In the current task context, shifting the exploration-exploitation trade-off toward greater exploitation would have reduced both uncertainty and cognitive demand, thereby streamlining decisional processing (Golubickis & Macrae, 2023).

The current findings extend the Self-Attention Network (SAN) model (Humphreys & Sui, 2016; Sui & Humphreys, 2015, 2017), which proposes that self-relevant information is prioritized through the coupling of perceptual and attentional systems, facilitating faster and more efficient processing of self-related (vs. other-related) stimuli. To date, support for this model has come primarily from perceptual matching and attentional cueing tasks (Sui et al., 2012; Sui & Rotsthein, 2019). The present results, however, suggest that this prioritization extends beyond perceptual facilitation to shape higher-order, value-based decision policies. That is, self-relevance alters not only how efficiently information is processed, but also how learned values are translated into action, shifting choice consistency (i.e., inverse temperature) and behavioral repetition (i.e., perseveration) in a temporally dynamic fashion. This suggests that the prioritized coupling of self-relevant information central to SAN may propagate downstream to influence decision-making under uncertainty, rather than being confined to early perceptual/attentional stages.

More broadly, the present findings inform theoretical accounts of learning by demonstrating that motivational factors linked to the self systematically guide how decision makers resolve the exploration-exploitation dilemma. Although reinforcement-learning frameworks have traditionally emphasized the role of environmental structure and computational constraints in governing the balance between reward maximization and uncertainty reduction (Cohen et al., 2007; Daw et al., 2006; Sutton & Barto, 1998), the current results indicate that this balance is also influenced by self-relevance. Across gain and loss contexts, self-related decisions were characterized by temporally dynamic but ultimately more deterministic choice patterns, consistent with a shift toward exploitation. These findings complement and extend prior work showing that self-relevance modulates reward sensitivity and learning rates (Golubickis & Macrae, 2022; Jalalian et al., 2024; Lockwood et al., 2022) by demonstrating that a primary influence of personal significance may lie in regulating the arbitration between competing decision policies. In so doing, the current results are consistent with emerging perspectives that conceptualize learning as an interaction between computational processes and motivational significance (Berger-Tal et al., 2014; Gershman, 2018), suggesting that models of adaptive behavior should incorporate self-relevance as a determinant of how individuals’ trade-off immediate reward against uncertainty reduction.

### Outstanding issues and future directions

As the first investigation of its kind, the current work yielded a set of interesting findings. In turn, these results naturally highlight several avenues for future research. Here, we outline three. First, the present findings suggest that motivational factors may influence resolution of the exploration-exploitation dilemma. It is therefore important to directly assess levels of self-enhancement motivation and acknowledge that individual differences in this construct are pertinent (Gregg et al., 2011), with personality characteristics and cultural factors playing a significant role. For instance, individuals with high self-esteem and traits such as narcissism or extraversion tend to exhibit stronger self-enhancing biases, whereas those with low self-esteem or high neuroticism display weaker tendencies (Hepper et al., 2010; John & Robbins, 1994; Sedikides & Gregg, 2008). Cultural context similarly shapes self-enhancement. Looming large in individualistic settings, self-enhancement tends to be downplayed in collectivist cultures in which self-criticism and modesty are deployed to preserve social harmony (Heine et al., 1999; Maddux et al., 2010). These individual and cultural differences have direct implications for the current findings, particularly regarding the extent to which such factors influence the decision policies underlying the exploration-exploitation dilemma. It should be noted, however, that the reliability of parameters estimated from computational models may be limited by a variety of factors (e.g., data quality, model assumptions, parameter uncertainty), thus should be interpreted with caution (Schaaf et al., 2024). Nevertheless, future work should examine these matters.

Second, the current findings underscore the role of the exploration-exploitation trade-off in surrogate decision-making (Batteux et al., 2019; Epley & Gilovich, 2016; Liu et al., 2018; Polman & Wu, 2020; Tunney & Ziegler, 2015). When making decisions for themselves, participants exhibited early exploitation in gain contexts and early exploration in loss contexts, reflecting strong engagement with personally salient outcomes. In contrast, decisions made for a friend were generally less extreme, with slower shifts in choice patterns, consistent with reduced motivational salience for others (Sedikides & Strube, 1997). Computational modeling indicated lower inverse temperature for friend-directed choices, suggesting more exploratory, less deterministic behavior. Relatedly, also suggestive of increased exploratory behavior, Liu et al. (2018) reported that when selecting a college course for someone else, participants sought more program-related information (e.g., pass rate, assignment difficulty) than when choosing for themselves (but see Liu & Baskin, 2021). Collectively, these findings suggest that self-enhancement and affective engagement systematically bias learning and choice policies, providing a framework for understanding when and why surrogate decision makers deviate from self-related strategies (Tunney & Ziegler, 2015). Given the importance of surrogate decision-making across domains such as healthcare and financial management, future research should probe and clarify the critical underlying computational mechanisms through which self-other differences emerge.

Third, an important challenge for future research will be to determine the extent to which the current findings generalize beyond restless MAB tasks to decision-making environments in which the explore-exploit dilemma is instantiated in richer and more structured ways. In this regard, foraging paradigms offer a promising avenue as they require individuals to decide not only which option to select, but also when to persist with or abandon a current resource patch (Charnov, 1976; Hayden et al., 2011; Kolling et al., 2012). Unlike bandit tasks, where options are discrete and simultaneously available, foraging environments impose sequential constraints and opportunity costs, making the valuation of alternatives inherently dynamic and context dependent. Examining the effects of self-relevance within such paradigms will enable researchers to test whether the observed increase in exploitative choice policies reflects a general tendency to prolong engagement with known resources when outcomes pertain to the self, or whether self-relevance instead modulates sensitivity to environmental variables such as patch depletion, travel costs, and background reward rates (Constantino & Daw, 2015; Le Heron et al., 2020). Additionally, foraging tasks provide a platform for integrating computational models of marginal value estimation with reinforcement-learning accounts, allowing a more precise characterization of how self-relevance influences both local decision policies and global environmental representations (Kolling et al., 2012; Mobbs et al., 2018; Niv et al., 2015).

### Conclusion

In sum, the present findings show that self-relevance systematically alters how individuals navigate the explore-exploit trade-off over time. Rather than exerting a static influence on performance, self-relevance dynamically modulates the extent to which value-based information guides choice, consistent with changes in underlying decision policies. These effects were observed across gain and loss contexts, suggesting a domain-general role for self-relevance in shaping adaptive behavior. By integrating time-resolved behavioral analyses with computational modeling, this work advances a nuanced account of how self-related processing influences value-based decisions.

## Data Availability

Available at https://osf.io/usgnx/overview?view_only=32bbefdda37f423bba65a17ea723d275.

## Declarations

### Public significance statement

Personal relevance modulates the temporal dynamics of learning, shaping how individuals navigate the explore-exploit trade-off. Ultimately, choices made for oneself are more consistent, reward-sensitive, and flexible than those made for others, revealing how self-relevance influences decision-making.
